# Evaluation of Selected Properties of Sodium Alginate-Based Hydrogel Material—Mechanical Strength, μDIC Analysis and Degradation

**DOI:** 10.3390/ma15031225

**Published:** 2022-02-06

**Authors:** Jagoda Kurowiak, Agnieszka Mackiewicz, Tomasz Klekiel, Romuald Będziński

**Affiliations:** Department of Biomedical Engineering, Institute of Material and Biomedical Engineering, Faculty of Mechanical Engineering, University of Zielona Góra, Licealna 9 Street, 65-417 Zielona Góra, Poland; a.mackiewicz@iimb.uz.zgora.pl (A.M.); t.klekiel@iimb.uz.zgora.pl (T.K.); r.bedzinski@iimb.uz.zgora.pl (R.B.)

**Keywords:** sodium alginate, hydrogels, stents, urology, digital image correlation

## Abstract

The search for ideal solutions for the treatment of urethral stenosis continues. This includes developing the material, design, while maintaining its optimal and desired properties. This paper presents the results of the research conducted on sodium alginate-based hydrogel material (AHM), which may be used as a material for stents dedicated to the treatment of pathologies occurring in the genitourinary system. In order to determine the selected parameters of the AHM samples, strength and degradation tests, as well as analysis of the micro changes occurring on the surface of the material using a digital image correlation (µDIC) system, were performed. This study shows that the material possessed good mechanical strength parameters, the knowledge of which is particularly important from the point of view of the stent-tissue interaction. The degradation analysis performed showed that the AHM samples degrade in an artificial urine environment, and that the degradation time mainly depends on the chemical composition of the material. The novel µDIC method performed allowed us to characterize the homogeneity of the material structure depending on the cross-linking agent used.

## 1. Introduction

Regenerative medicine, especially today, is one of the most important fields in interdisciplinary sciences, combining the knowledge of medicine, chemistry, biology and biomedical engineering. It allows to strive for improvement and progress in the regeneration of damaged tissues or possible replacement of organs. Its wide application is a result of virtually unlimited possibilities offered by the progress in tissue engineering research, new stem cell technologies, discovery of new combinations of biomaterials based on natural or synthetic polymers and the improvement of existing solutions. The task of biomaterials used in regenerative medicine is to form a scaffold with the cells of the body or tissues, allowing the start of the formation process of new cells for the tissue regeneration [1,2,3,4,5,6,7,8].

The development of regenerative medicine is also very important from the point of view of the increasingly diagnosed diseases of the genitourinary system. The urethra, which is a part of this system, is a tubular structure consisting of several soft tissue layers. Its structure is similar to the bladder. The primary function of the urethra is to drain the urine from the body. The male urethra is significantly longer than the female urethra. The difference in length is due to the anatomical structure of the body. In the case of men, the urethra also has the function of carrying out semen. Due to the fact that the male urethra is longer and is approximately 15–20 cm, it is exposed to diseases much more frequently [9,10,11]. 

A common condition among men is urethral stricture. Urethral stricture is caused by scarring that results from the replacement of spongy tissue by scar tissue. The transformation of the tissue is most often caused by external irritants or previous surgical interventions. The sooner stenosis is diagnosed, the more effective its treatment can be. It is important to note that, if left untreated, this condition can lead to serious problems that impair the lower urinary tract, including micturition disorders or impaired kidney function. The treatment methods used so far, especially those requiring advanced surgical interventions such as urethrotomy or urethroplasty, work only in isolated cases involving a short stricture segment and are not an effective treatment for recurrent strictures with extensive inflammatory reaction [12]. Another technique that has been used for a long time is urethral calibration, which involves inserting dilators of increasing diameter into the narrowed areas to stretch the urethral tissue wall. However, its use gives short-term results and requires periodic repetition, which can lead to serious postoperative complications, such as bacterial infections [13,14]. Hence, the need for further research to develop a method that allows for a long-term cure without the need of secondary surgical interventions. 

Currently, the most popular treating method used for the narrowing of the urethral canal is stenting, which has systematically been improved by introducing new solutions. Until now, metal or aluminum stents have been the most commonly used [15,16,17], but, today, researchers are looking for better solutions, including those based on the stents from biodegradable materials [18,19,20,21,22,23,24]. The evolution of stents dedicated to the genitourinary system for both the ureter and the urethra has been very intense. The first attempts to remove the obstruction from the urinary drainage channel consisted of simple constructions made of leaves, reeds or straws [25]. Subsequently, attempts to catheterize were made using flexible catheters made of animal skin. With the discovery of new technologies and processes, some of the first flexible stents were rubber-based. Together with the development of science, catheters or personalized stents were introduced [25,26]. Currently, the stents are considered a standard and essential tool in the treatment of urethra abnormalities. However, it should be noted that despite significant progress in both the materials used for stents and their design, they do not yet meet the requirements for “ideal stents”. Ideal stents should be primarily characterized by non-migration, biocompatibility with the surrounding tissue, should not cause encrustation and should preserve urinary flow trajectories [27,28]. It remains a challenge to find the best solution that is medically ideal and well tolerated inside the body.

Urological stents should be characterized by high deformability, with mechanical characteristics similar to the tissues of the genitourinary system. Differences in the mechanical characteristics of tissues may result from the species of an individual, its age, race or body weight, but also from the method of determination: an experiment or numerical modeling [29]. In case of the human urethra, the value of Young’s modulus, depending on the research method, is 0.3 MPa (experimental studies) presented in Zhang et al. [30], 2.4 MPa (experimental studies) presented in Yao et al. [31], 5 MPa (experimental studies) presented in Spirka et al. [32]. The urethra of a rabbits shows the value of Young’s modulus: 0.25–0.5 MPa (experimental studies) presented in Feng, et.al., and Zhang, et.al. [33,34].

One of the bioresorbable materials of urological stents is sodium alginate (SA) [3,21,22,35,36]. Hydrogel materials based on sodium alginate are primarily bio- and cytocompatible, biodegradable and exhibit antimicrobial properties. Due to their properties, they are widely used for stent manufacturing, in drug delivery systems or as scaffolds for tissue engineering [8,37,38,39,40,41,42,43]. SA belongs to the group of natural polymers and is an anionic and hydrophilic polysaccharide. It is mainly extracted from brown seaweed. It is composed of β-D-mannuronic acid (M) and α-L-guluronic acid (G) monomer blocks. The stacking structure of the blocks can occur in three configurations: segments of G residues, M residues or alternating M–G residues. The unique properties of sodium alginate include its biocompatibility, biodegradability and lack of antigenicity and toxicity to the organism and relative ease in gel formation with divalent cations, most commonly Ca^2+^ [38,44,45]. Divalent Ca^2+^ cations used for cross-linking and gel formation, although the most popular, also have disadvantages. Calcium tends to form calcifications within tissues, which can cause additional clogging of the flow lumen. As a result, the research is being conducted into other elements that will have similar properties and will not be toxic to the body. For the analyzed hydrogel material (AHM) based on the SA, the mechanical properties can be changed by the number and sequence of M and G blocks. A higher number of G blocks provides higher stiffness and strength of the material. The gelation time has influence on homogeneity of structures and increases the elasticity [46].

Any study of hydrogel materials used in the tissue environment is concentrated on determination of the material characteristics. The degree of degradation and the mechanical strength, which should be as close to deformability of the tissue as possible, are also determined. Degradation studies and static tensile testing are popular methods for testing hydrogel materials. 

An innovation in the current study is that an attempt has been made to perform hydrogel testing using a micro digital image correlation (µDIC) system. This new methodology is carried out by the gradual expansion of the diameter of the stent made of AHM using a cardiac balloon. The 3D µDIC technique uses two cameras that are positioned on the surface of an object at the correct angle for the required field of view. A calibration target is then used to allow the calculation and reconstruction of the 3D space from each of the 2D views from the two cameras. To perform µDIC measurements, it is necessary to have a stochastic pattern (random dots) on the sample so that the correlation algorithm can track the displacement or movement of the surface as it deforms. Using this information allows the 3D deformation and the resulting strain field to be calculated from sequential images of the object. This technique allows for non-contact measurement of deformation which allows for real results of contour changes and surface deformation, including micro-scale. The study of micro-deformation and contour changes can be performed for specimens of various shapes, even highly irregular ones; however, before the measurements are made, the camera-lens system is calibrated by imaging elements with a known separation length (i.e., the calibration target) [47,48]. In the case of our study, tubular-shaped specimens were used due to their intended use as a urological stent for urethra obstruction. The determined mechanical behavior characteristics of different materials are necessary and are a fundamental part of the assessment of strength and proper operation of mechanical systems inside the body determined in the biomedical research. Mechanical testing was performed to compare the samples and to identify their constitutive strength parameters. However, some discrepancies in the results, which arise due to average strain measures, heterogeneity of the material geometry and structure and specimen assembly in the measurement system, are unavoidable. In order to refine and accurately assess the mechanical performance of the specimen, local deformation tests can be performed on the material. Evaluation of local surface deformation, in this case of a sodium alginate-based hydrogel material, can be performed on a device equipped with high-resolution cameras and digital image correlation technology. Image series of the sample surface under uniaxial incremental loading are obtained to determine local deformation. The µDIC technique can greatly improve the accuracy of experimental studies, not only of hydrogel or polymeric materials, but also of soft tissues [49,50,51,52]. In this study, the aim of the investigation is to determine the material characteristics of AHM cross-linked with divalent Ca^2+^ and Ba^2+^ cations and to determine how the parameters of AHM.

## 2. Materials and Methods

Material used for the stents should be properly selected, especially regarding particular conditions. These conditions are pressure, temperature, humidity and chemical environment. Furthermore, the stents should allow for the influence of the muscles. The uniaxial tensile test, degradation analysis, and structural evaluation are required to right selected of the material.

### 2.1. Materials

Tests were carried out using salt of alginate acid (Sigma-Aldrich, Poznan, Poland) with the following parameters: viscosity 15–25 cP, 1% H_2_O, pH 6.5–8.5, molecular weight 120–190 g/mol and density 1.601 g/cm^3^ and calcium chloride CaCl_2_ and barium chloride BaCl_2_ (Sigma-Aldrich, Poznan, Poland). Chemical reagents needed for the preparation of artificial urine solution according to Mayrovitz and Sims [53]: urea (Sigma-Aldrich, Poznan, Poland), creatinine (Sigma-Aldrich, Poznan, Poland), sodium chloride NaCl (Sigma-Aldrich, Poznan, Poland), ammonium chloride NH_4_Cl (Chempur, Poland), sodium sulfate Na_2_SO_4_ (Avantor Performance Materials Poland S.A., Gliwice, Poland) and di-sodium phosphate (Avantor Performance Materials Poland S.A., Gliwice, Poland). All the solutions were prepared in deionized water.

### 2.2. Sample Preparation

Samples with different contents of SA dissolved in 100 mL of deionized water (5 g/100 mL or 7 g/100 mL) cross-linked with 1.5 M calcium chloride solution and barium chloride were prepared. Degradation and tensile strength tests using a testing machine and a digital image correlation system (µDIC) were conducted on tubular specimens; the shape was selected as similar to the stent. The sample preparation recipe of SA was described in the previous studies [3,22]. Briefly, the alginate acid salt was dissolved for 16 h in 100 mL of deionized water until the powder dissolved in an electromagnetic stirrer, then air bubbles were removed from the solution in an ultrasonic washer. Calcium chloride and barium chloride at the concentration of 1.5 M were dissolved in 100 mL of deionized water for a minimum of 2 h. The solutions were prepared at ambient conditions of 23 °C. The test specimens were prepared with the sol-gel method in the shape of tubes, taking the sequence of dipping: alginate, then cross-linking substance. Forming the tubular samples using the sol-gel method initially took 2 min in each solution, which allowed for the initial shaping of the stent. Then, the samples were divided into four groups due to different cross-linking times (Table 1): I—cross-linking time 2 h, II—cross-linking time 24 h, III—cross-linking time 48 h and IV—cross-linking time 72 h. The samples were then immersed in the cross-linking substance in order to obtain the maximally saturated egg-box structure of the material. The purpose of using different cross-linking periods of time was to determine whether and, if necessary, how the parameters of the material properties of the alginate/CaCl_2_ and alginate/BaCl_2_ connections changed.

### 2.3. Stiffness of the Material

**Figure 1:**
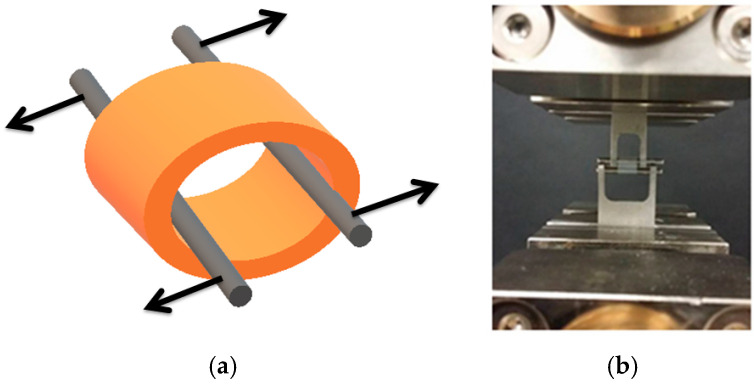
Strength testing of hydrogel samples: (**a**) schematic representation of tests conducted, (**b**) in-house experimental studies.

The tensile test was realized using a Zwick Roell EPZ 005 testing machine (Zwick Roell, Ulm, Germany). Tube-shaped specimens (Figure 1) with average dimensions of wall thickness 0.6 ÷ 0.7 mm, outer diameter 3.8 ÷ 5.5 mm, and length 3.5 ÷ 6 mm, were subjected to the static tensile testing. For each type of hydrogel material, tests were performed on three samples (16 types of hydrogel material × 3 samples). The result was used to determine its elasticity (Young’s modulus). The samples differed in the cross-linking time and the type of Ca^2+^ or Ba^2+^ cross-linking cations.

### 2.4. Material Micro-Deformation Analysis Using µDIC

**Figure 2:**
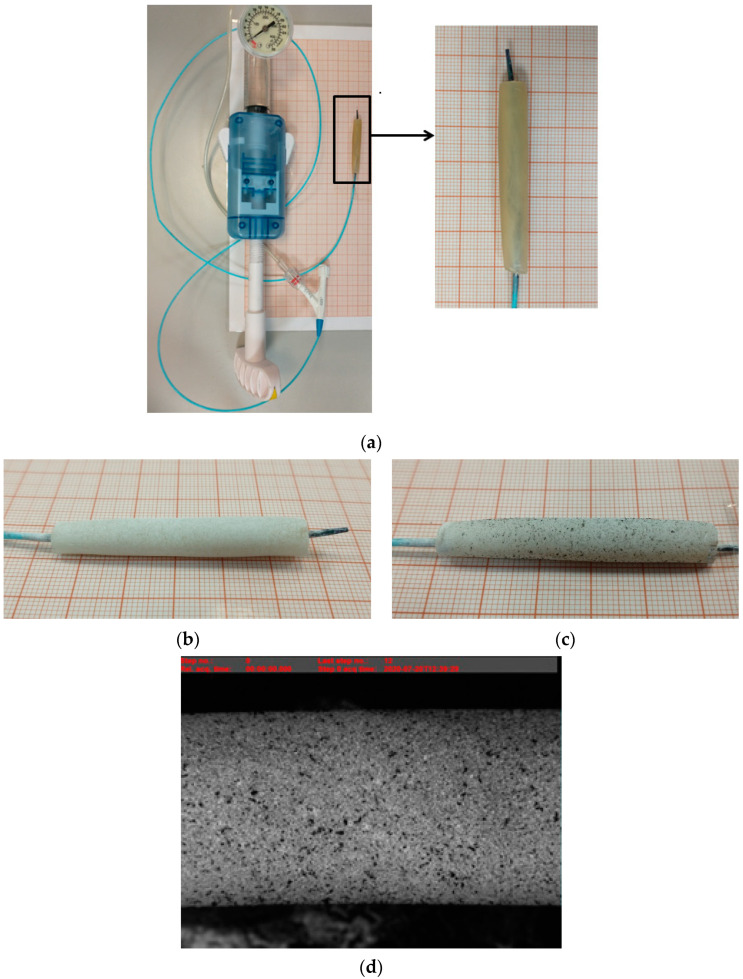
Example of sample preparation for µDIC study: (**a**) AHM placed on a cardiac balloon, (**b**) matted AHM sample, (**c**) AHM sample with pattern applied—activated carbon, (**d**) example image of an AHM sample obtained with the µDIC system.

The µDIC allows the recording and observation of changes occurring in the material structure at the micro accuracy under load realized by external forces. The micro displacement measurement is defined as the change in surface coordinates resulting from an applied forcing due to balloon expansion. To make the measurement, a stochastic pattern is applied to the sample surface. A sequence of digital images for successive loading steps allows for the detection of changes in the three-dimensional position of these points, showing micro displacement maps whose values correspond to the corresponding colors shown in the scale adjacent to the figure. The study consisted of progressively increasing the diameter of the stent samples using a cardiac balloon. The tubular samples prepared for µDIC tests had to be slightly dried from water using filter paper; then, the material was matted and, finally, the pattern in the form of activated carbon was applied (Figure 2). The activated carbon particles were applied to the hydrogel material at a distance of about 15–20 cm from the surface of the sample. The samples prepared in this way could be mounted on the test stand. The samples with an outer diameter of 3.8–5.6 mm and a length of 35–40 mm were placed on an uninflated balloon. Then, deionized water was pumped into the balloon until the maximum diameter of the balloon reached 8 mm. Changes in the form of increasing stent diameter were recorded by taking a photograph of every half-revolution of the syringe pump. The µDIC tests allowed for the assessment of deformations, occurring in the analyzed material with division into particular groups. For each type of hydrogel material, tests were performed on three samples (16 types of hydrogel material × 3 samples). The surface observation allowed checking how particular samples behave and the changes on the material surface show the level of the structure homogeneity.

### 2.5. In Vitro Degradation Test

**Figure 3:**
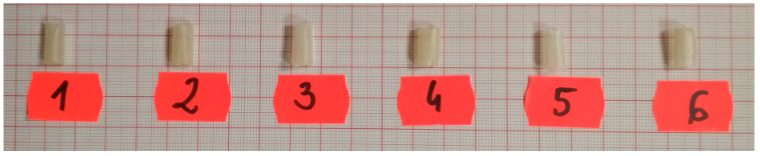
Example tubular samples produced by sol-gel method for degradation studies.

Degradation of the material was carried out similarly to the studies [3,21]. The artificial urine solution was prepared according to the recipe [53]. Sodium alginate-based hydrogel material of 0.05 g/mL or 0.07 g/mL cross-linked with 1.5 M CaCl_2_ or BaCl_2_ solution was subjected to in vitro degradation studies. Degradation by weight was carried out by complete and continuous immersion of the material in a 20 mL artificial urine solution for 1, 3, 5 and 7 days. After each immersion, the sample was dried at room temperature for 24 h. The dimensions of most samples were within an outer diameter from 4 to 6 mm and a length from 10 to 13 mm. Example samples are presented in Figure 3. For each type of hydrogel material, degradation tests were performed on two samples (16 types of hydrogel material × 2 samples). Tubular samples were manufactured for testing.

Degradation studies were conducted at 37 °C in order to mimic the temperature conditions found in the urethral tissue as closely as possible. The study was designed to determine what effect on the degradation time caused in different concentrations of sodium alginate, type of divalent cation and cross-linking time used. Degradation was calculated based on Equation (1):

Degradation % = ((Wwet − Wdry)/Wwet) × 100%,(1)

where, Wwet—initial weight of the sample, Wdry—weight of the sample dried after immersion in artificial urine solution. 

## 3. Results and Discussion

### 3.1. Stiffness of the Material

**Figure 4:**
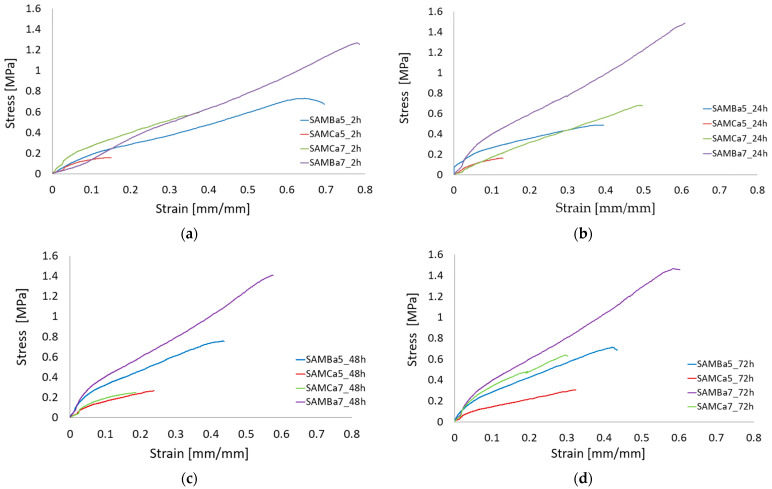
Strain-strain analysis plot for the proposed sodium alginate based material cross-linked with Ca^2+^ or Ba^2+^ cations using different cross-linking time: (**a**) 2 h cross-linking time, (**b**) 24 h cross-linking time, (**c**) 4 h cross-linking time, (**d**) 72 h cross-linking time.

**Figure 5:**
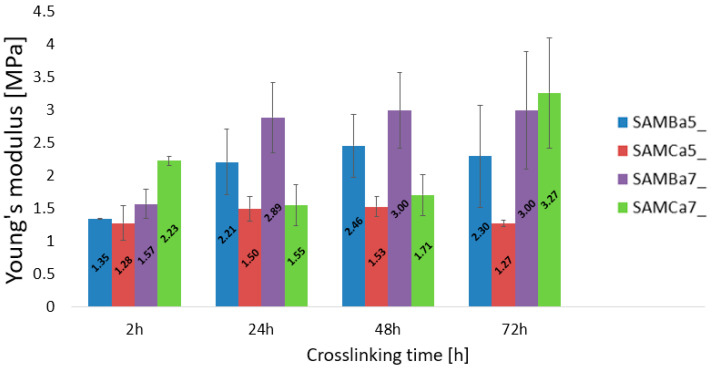
Young’s modulus of the test material for different cross-linking times: 2 h, 24 h, 48 h, 72 h.

The uniaxial tensile tests performed on the tubular hydrogel materials are presented in Figure 4 and Figure 5. The results state how the material characteristics change for varied values of concentration and cross-linking time. Figure 4 a–d shows the stress–strain changed for four groups of materials: I—cross-linking time 2 h, II—cross-linking time 24 h, III—cross-linking time 48 h and IV—cross-linking time 72 h. The graphs show that the samples with the alginate content of 0.05 g/mL and 0.07 g/mL cross-linked with 1.5 molar barium chloride at different cross-linking times were characterized by the best repeatability. The strain level of the sample, as well as the strength value for the sample (maximum stress value) with 0.05 g/mL and 0.07 g/mL alginate content crosslinked with 1.5 mol barium chloride at different crosslinking times are similar to each other, which is not seen for other variants. The studies showed that the hydrogel material crosslinked with Ca^2+^ cations was characterized by lower mechanical strength than that of the material crosslinked with Ba^2+^ cations. A relation saying that mechanical properties of hydrogels crosslinked with calcium change depending on crosslinking time was also observed. As presented by Mørch et al., the affinity of alginate to divalent cations decreases according to the following order: Pb > Cu > Cd > Ba > Sr > Ca > Co, Ni, Zn > Mn. Literature reports indicate that, in physiological solutions (liquids), magnesium and sodium ions in contact with the material cause its swelling, which leads to destabilization and disruption of hydrogel bonds. The obtained results of our study indicate the confirmation of the hypothesis that barium shows greater affinity to alginate. Thus, it forms gels with a stronger structure than those crosslinked with calcium cations [54]. The G blocks together with divalent cations form the egg-box structure, which is necessary for the material polymerization [55].

The elasticity properties of the tested samples are presented in Figure 5. The sample SAMBa7 is independent to cross-linking times and is characterized by the best stability. It turns out that even 24-h curing of the sample with 7 g/100 mL of sodium alginate cross-linked with 1.5 mol BaCl_2_ solution is sufficient for the material to reach a high value of Young’s modulus, oscillating around 3 MPa. Samples with an alginate content of 0.05 g/mL crosslinked with calcium or barium show relatively reproducible values regardless of crosslinking time. The material containing 0.05 g/mL of sodium alginate crosslinked with 1.5 mol CaCl_2_ solution proved to be the most unstable. The results obtained, in comparison to the other investigations [46,56,57], are not the same. This is due to, among other things, the use of different crosslinking times (minutes, hours, or days), different concentrations of crosslinking agent, different concentrations of hydrogel solution, the use of additives to the starting hydrogel, and the method of materials preparation itself. Applying changes to even one of these conditions can produce different results, which can sometimes allow for improved mechanical properties. The lowest strain value from 0.15 to 0.3 mm/mm and the lowest strength value from 0.1 to 0.2 MPa turned out to belong to the one containing 0.05 g/mL of sodium alginate crosslinked with 1.5 mol CaCl_2_ solution. 

### 3.2. Material Micro-Deformation Analysis Using µDIC Digital Image Correlation

**Figure 6:**
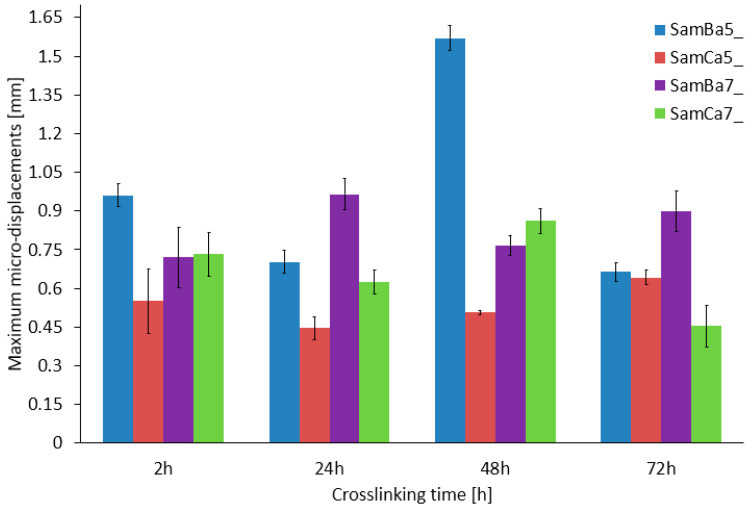
Maximum micro displacements tested for the samples analyzed.

Micro-displacement and micro-deformation studies using a digital image correlation system (µDIC System, Dantec Dynamic, Denmark), were performed to determine three-dimensional surface changes in the form of structural discontinuities, (Figure 6) occurring in the analyzed material with division into particular groups. For each type of hydrogel material, tests were performed on three samples (16 types of hydrogel material × 3 samples).

**Figure 7:**
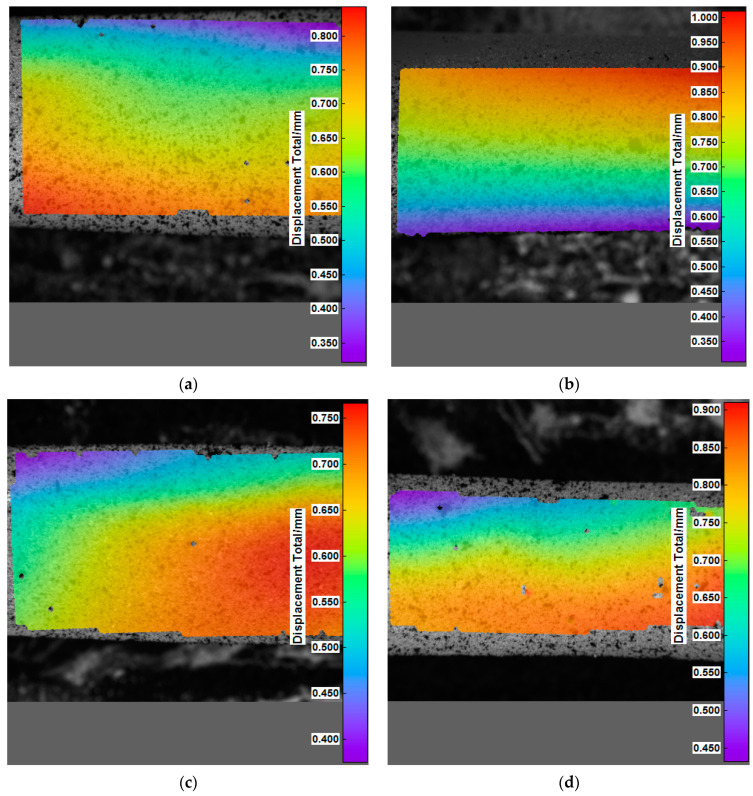
Example analysis of micro-displacement distribution for the tested material SamBa7_2h (**a**), SamBa7_24h (**b**), SamBa7_48h (**c**), SamBa7_72h (**d**).

The study shows that changes in the material micro-displacement occurring as a result of controlled increasing of the diameter depended on the cross-linking agent and the cross-linking time. For the samples cross-linked with Ca^2+^ cations, it was observed that the micro displacements for different cross-linking times were similar and relatively constant, ranging from 0.45–0.82 mm. On the other hand, the results of the samples cross-linked with Ba^2+^ cations are characterized by a slightly higher variation, in which the micro displacements are in the range of 0.85–1.6 mm. A significant differentiation was observed in the material with the alginate content equal to 0.05 g/mL Ba^2+^ cross-linked. The samples with 0.07 g/mL alginate crosslinked with Ba^2+^ (Figure 7) showed maximum micro displacements, ranging from about 0.75 to 1.0 mm for all crosslinking times, which is the best result with respect to the other sample types, with no features of large scatter in the final values.

The analysis of the µDIC results shown that the barium chloride cross-linked material had more reproducible results. This material shows more elasticity and stiffness in comparison to the calcium cross-linked samples. Longer cross-linking time increased the stiffness. In the case of calcium cross-linked samples, weaker cross-linking of the structure was observed, which caused considerable discrepancies in the results obtained for these samples. It is also worth mentioning that SAMBa5_24h and SAMBa7_48h showed the highest structural stability. It was observed that the samples cross-linked with calcium chloride were more susceptible to damage just by mounting the material on a cardiac balloon.

### 3.3. In Vitro Degradation Test

**Figure 8:**
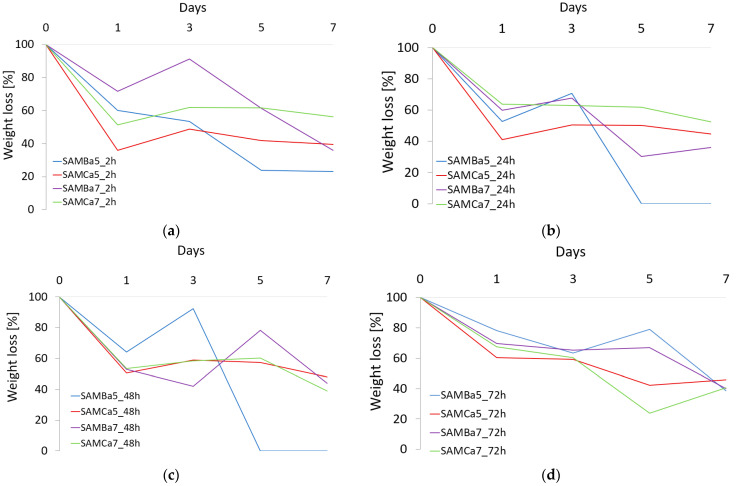
Degradation studies for the proposed material based on sodium alginate cross-linked with Ca^2+^ or Ba^2+^ cations using different cross-linking time: (**a**) 2 h cross-linking time, (**b**) 24 h cross-linking time, (**c**) 48 h cross-linking time, (**d**) 72 h cross-linking time.

To evaluate the AHM, the degradation in artificial urine solution was performed according to AU-Maurowitz recipe [53]. The analysis was carried out for a period of 7 days or until complete degradation of the material. Figure 8 shows the results obtained for four groups of materials.

Analyzing each of the four groups of materials, I—2 h cross-linking time, II—24 h cross-linking time, III—48 h cross-linking time and IV—72 h cross-linking time, it was found that the SAMBa5 sample degraded the fastest. The sample with the alginate content of 0.05 g/mL cross-linked with Ba^2+^ cations for 24 and 48 h on the 5th day of the test showed complete degradation. It was observed that the degradation rate of the samples cross-linked with Ca^2+^ cations for all cross-linking times (2, 24, 48 and 72 h), was relatively constant and amounted to 40–55% by the 7th day of the test. Samples cross-linked with Ba^2+^ cations are more variable. The occurrence of swelling phenomenon in some of the samples is due to the penetration and replacement of free bonds or gaps after cation atoms by H_2_O molecules [3]. The degradation results obtained for the samples cross-linked with divalent Ca^2+^ cations are similar to those reported in other investigations [21,40,57]. Importantly, during the course of the study, significant changes were observed in the structural behavior of different material types after contact with the artificial urine solution. Prior to the contact of the material with the artificial urine solution, mainly the samples with different sodium alginate content but cross-linked with 1.5 mol BaCl_2_ solution were stiffer and more durable than the samples cross-linked with 1.5 mol CaCl_2_ solution. However, it appeared that the samples cross-linked with divalent Ca^2+^ cations were more resistant and durable during the contact with the artificial urine solution. The samples cross-linked with divalent Ba^2+^ cations degraded faster; their material characteristics changed as early as the first day of contact with urine. For example, the SAMCa_7 sample, unlike the SAMBa_7 sample, was not completely distorted by a collapse of the stent wall. Degradation studies of hydrogel materials are primarily characterized by the disruption of polymer chains, leading to a reduction in molecular weight. This is accompanied by the formation of macromolecules with lower molecular weights, with the formation of branched structures. The number and position of bonds also change. In the conducted studies, the moments of hydrogel swelling were observed on day 3, which is shown in Figure 8. This is most likely due to the penetration and replacement of free bonds and cationic sites with water molecules, especially in the deeper layers of the hydrogel. This phenomenon is the result of contact between the hydrogel material and the artificial urine solution, where initial and partial degradation occurred after immersion, which exposed the deeper layers of unreacted bonds. 

## 4. Conclusions

Observation of the samples of AHM cross-linked with Ba^2+^ cations proved that they showed better mechanical properties than those cross-linked with Ca^2+^ cations. 24 h cross-linking of AHM was sufficient to achieve maximum exchange between the polymer G-blocks and cross-linking cations (egg-box structure). Application of the µDIC technique allowed for the determination of the micro-changes occurring in the structure of the hydrogel material, which is susceptible to deformation due to external forces and homogeneity structure. The hydrogel variants investigated are dedicated as a material for the fabrication of stents to decongest fibrotic urethral tissue. To insert the finished tubular structure into the urethra canal, the authors suggest using a balloon on which the diameter of the stent can be increased and expanded by balloon expansion. As a result of the increased inner diameter of the tubular specimen, it can successfully remain inside the urethra. Additionally, it is important to note that, during the flow of urine, the urethra deforms in the radial direction, increasing the lumen diameter, such as in the balloon test and µDIC imaging we performed. The performed tests are innovative and allow precise determination of contour changes and surface structure deformation; hence, it should be classified, opposite to full detailed research, as a scientific experiment for which, usually, the statistical analysis is not made and the experiment is realized for a small number of samples. µDIC analysis of the displacements induced by increasing the diameter of a stent with a cardiac balloon showed that the samples cross-linked with Ba^2+^ are more flexible than those cross-linked with Ca^2+^ cations. Additionally, increasing the diameter of the material for the samples cross-linked with Ca^2+^ cations was technically more difficult due to very vulnerable structure of the material, which is most likely due to the chemically weaker egg-box structure, Ca^2+^ cations have lower atomic mass than Ba^2+^ cations. Finally, the results presented in this article led to the conclusion that degradation studies showed that the sodium alginate-based hydrogel material degraded approximately 60–70% regardless of the cross-linking time during 7 days of continuous immersion in the artificial urine solution. Optimal material parameters, based on which it is possible to construct a stent with the controlled resorption time, were achieved. These properties are particularly important because they may promote the treatment of inflammatory conditions of the urethra. The applied methods of the material evaluation gave a full picture of the mechanical and physical properties of these materials. The presented methodology can be successfully used in the analysis of similar research and issues.

## Figures and Tables

**Table 1:** Names and basic parameters of stents prepared with sodium alginate.

Sample Name	Concentration of Sodium Alginate [g/mL]	Concentration of Crosslinking Agent [mol]	Crosslinking Time [h]
SAMBa5_2h	0.05	1.5	2
SAMCa5_2h			
SAMBa5_24h			24
SAMCa5_24h			
SAMBa5_48h			48
SAMCa5_48h			
SAMBa5_72h			72
SAMCa5_72h			
SAMBa7_2h	0.07		2
SAMCa7_2h			
SAMBa7_24h			24
SAMCa7_24h			
SAMBa7_48h			48
SAMCa7_48h			
SAMBa7_72h			72
SAMCa7_72h			

## Data Availability

Not applicable.
